# Clinical supervision approach predicts evidence-based trauma treatment delivery in children’s mental health

**DOI:** 10.3389/fpsyt.2022.1072844

**Published:** 2023-01-09

**Authors:** Rosemary D. Meza, Rashed AlRasheed, Michael D. Pullmann, Shannon Dorsey

**Affiliations:** ^1^Kaiser Permanente Washington Health Research Institute, Seattle, WA, United States; ^2^Department of Psychology, University of Washington, Seattle, WA, United States; ^3^Department of Psychiatry and Behavioral Sciences, University of Washington, Seattle, WA, United States

**Keywords:** workplace-based supervision, evidence-based treatment, treatment delivery, community mental health, children and adolescent

## Abstract

**Objective:**

Observational studies of practices used in clinical supervision-as-usual can be leveraged to advance the limited research on workplace-based supervision as an evidence-based treatment (EBT) implementation strategy. This exploratory observational study examined the presence of supervision approaches (comprised of supervision techniques) and whether these predicted clinicians’ EBT technique delivery.

**Methods:**

Participants included 28 supervisors, 70 clinician supervisees, and 60 youth clients and guardians from 17 public mental health organizations. Data included audio recorded supervision-as-usual sessions over 1 year, audio recorded Trauma-focused Cognitive Behavioral Therapy (TF-CBT) treatment sessions with youth for 6 months, and youth-reported post-traumatic stress severity scores. Audio recordings of 438 supervision sessions were coded for session duration and the presence of 13 supervision techniques and intensity of their coverage. Audio recordings of 465 treatment sessions were coded for presence and intensity of coverage of TF-CBT practice elements. Agglomerative hierarchical cluster analysis examined the presence of clusters of supervision technique use, termed supervision approaches. Generalized estimating equations estimated the relation between supervision approaches and delivery of TF-CBT elements.

**Results:**

Two supervision approaches were identified– *Supportive*–*Directive* and *Supportive*– that discriminated between use of five supervision techniques. Clinicians who received a higher proportion of *supportive*–*directive* supervision sessions had greater odds of delivering the trauma narrative with a client.

**Conclusion:**

Findings suggest that patterns of supervision techniques can be identified and may shape EBT delivery. Supervision approaches show some evidence of being tailored to individual clinicians. Implications for the development of supervision implementation strategies and future directions are discussed.

## 1. Introduction

Evidence suggests that clinical supervision can positively impact providers’ evidence-based treatment (EBT) delivery (1) and client outcomes (2, 3). Clinical supervision is carried out by a senior clinician with a more junior clinician, is evaluative in nature, ongoing, and serves to enhance clinician’s knowledge, competency, autonomy and ensure service quality (4). It is the foremost method by which mental health professionals receive training in therapeutic practices (5) and is a necessary support to positively impact providers’ behavior after receiving training in an EBT (6). Clinical supervision focused on EBT has been shown to improve clinicians’ EBT attitudes, knowledge, skills, and fidelity (1, 7). Despite its importance, workplace-supervision provided by in-house supervisors remains an understudied implementation strategy (2)—a method to support the adoption and sustained use of EBTs (8). Clinical supervision holds promise as a feasible and cost-effective implementation strategy as it is commonly available in community mental health settings (9). Despite broad availability in many settings, supervision time is limited with estimates of community mental health clinicians receiving an average of approximately 1−2 h weekly, consisting of clinical and non-clinical topics (10, 11). This reflects an estimated 5−12 mins on average per case weekly (11, 12), although it is likely a few cases receive the bulk of supervision time. Descriptive studies of how supervision time is spent suggest that limited time is focused on content most relevant to EBTs and when it is discussed, it is rarely thoroughly discussed (10, 13). Given these constraints on workplace-based supervision, a clear understanding of the specific techniques used in supervision and how those relate to clinicians’ practice is needed. By understanding how supervision technique use relates to the delivery of EBTs, we can inform efforts aimed at enhancing access to and quality of treatment for youth being served in community-based settings.

Techniques proposed in supervision models and those used in efficacy trials, EBT training studies, and EBT consultation studies provide a foundation for the techniques that may be employed in workplace-based supervision to impact providers treatment behaviors. At a broad level, Milne (4) proposed that clinical competence can be developed through integration of four components of the experiential learning cycle during supervision: experiencing, reflecting, conceptualizing, and planning. Efficacy trials of clinical interventions typically use a common set of “gold standard” supervision techniques including skill-building/behavioral rehearsal, fidelity monitoring, live or recorded review of practice, and client symptom monitoring (14, 15). A review of studies examining EBT training methods suggests that passive didactic methods of training (e.g., presentations) have limited effects on behavior, and active learning strategies are required to influence therapists behavior and client change (6). Active learning strategies require the trainee to participate in the skill being taught (e.g., modeling, behavioral rehearsals, and coaching and feedback). A systematic review of supervision practices associated with formative outcomes, such a provider skill development and knowledge, found that corrective feedback, discussing intervention, behavioral rehearsal, case conceptualization, agenda setting, live corrective feedback, modeling, and empathy were the most common supervision strategies included in supervision that was associated with improvements in a formative outcome (16). These bodies of literature suggest numerous supervision techniques that may be used in workplace-based supervision to support clinicians in delivering EBT, broadly encompassing directive teaching, experiential learning, feedback, client and practice monitoring, and reflection.

Two studies of workplace-based EBT supervision have tested EBT-specific models of supervision that incorporate gold-standard techniques and found positive effects on clinician adherence and client outcomes. An observational study of Multisystemic Therapy (MST) across 45 youth-serving organization in 12 states and Canada found that supervisor adherence to a manualized MST supervision approach predicted improved clinician adherence (1) and child outcomes (2). Another randomized control trial compared supervision as usual to a Motivational Interviewing (MI) supervision approach to supervise clinician delivery of MI (17). MI supervision included corrective feedback based on review of actual practice and skills coaching using behavioral rehearsal. Clinicians in the MI supervision condition demonstrated greater competency in MI compared to those that received supervision as usual. These studies provide support for the importance of techniques involving practice, observation, feedback, and fidelity monitoring in impacting clinician adherence, competency, and client outcomes in the context of workplace-based supervision. While these studies were conducted in routine practice settings, supervisors received extensive support to shape their practice, such as training from expert supervisors, observation of their supervision and corrective feedback, and ongoing consultation. These studies contribute to the evidence that supervision can influence important outcomes, but there remain gaps in the use of gold-standard supervision techniques in naturally occurring workplace-based supervision.

Recent descriptive studies of workplace-based supervision have identified gaps in the use of gold-standard and evidence-based supervision techniques. One study measured the presence and intensity of supervision techniques used during supervision in the context of a state-funded implementation of Trauma-Focused Cognitive Behavioral Therapy (TF-CBT) in community mental health organizations (12). The most frequently used techniques in supervision, and often used with medium to high intensity, included supportive listening, information gathering, didactic instruction, providing clinical suggestions and fidelity/adherence assessment. Eight of the thirteen techniques were used with mostly low intensity, including supervision techniques commonly used in treatment trials (e.g., review of actual practice, behavioral rehearsal). Bailin et al. (18) measured the frequency, duration, and competency (i.e., skillfulness of delivery ranging from superficial to expert) of supervision content and techniques, which they term “micro-skills,” present in supervision for clinicians treating youth with a variety of problem areas (18). Among micro-skills considered to be evidence-based (i.e., corrective feedback, modeling, and behavioral rehearsal), there was significant variability in how often they were used and the time allocated to their use. For instance, modeling was used in 70.2% of sessions, while behavioral rehearsal was used in 1.8% of sessions. The competency with which these micro-skills were delivered also tended to be low. Other micro-skills that occurred frequently and were allotted significant time included administrative tasks, case conceptualization, praise, and supervisor self-disclosure.

These descriptive studies identify discrepancies between “gold standard” techniques and those used in workplace-based supervision. Across categories of techniques, the studies found high-to-moderate use of directive techniques (e.g., didactic instruction and clinical suggestions), infrequent or poor competence with experiential techniques (e.g., behavioral rehearsal and modeling), and frequent passive supportive techniques (e.g., supportive listening and empathy). The use of monitoring and quality assurance techniques (e.g., symptom monitoring and reviewing actual practice) were mixed, with symptom monitoring being common, but review of practice occurring rarely. These discrepancies in the use of gold standard techniques could be due to a complex array of factors, ranging from the broad set of functions that supervision fulfill that could limit time for gold-standard techniques (10) to characteristics of the clinicians, supervisors or settings in which they work. For instance, most supervisors do not have formal training in supervision (19), which may limit their knowledge of “gold standard” techniques. Other plausible contributors to discrepancies include features of supervisors’ educational background and their theoretical orientation. Bailin et al. (18) found that doctoral level supervisors used modeling with greater competence than master’s level supervisors and supervisors in public mental health allotted more time to and used modeling with greater competency relative to those in private settings. However, they found no link between a supervisor’s theoretical orientation and the delivery of any evidence-based micro-skills.

While the reviewed literature demonstrates that on average evidence-based supervision techniques are used infrequently and often with low competency (12, 18), they do not capture how supervision techniques are used in combination. Rather than operating independently, supervision techniques are likely used in combination to complement one another. The current study advances our understanding of workplace-based supervision by investigating how supervision techniques are used in combination with one another to comprise a supervision approach. Additionally, this study explores the extent to which supervision approaches vary from session to session and by the clinician being supervised. By characterizing this variability, we can begin to inform hypotheses about contextual factors, such as time available for supervision, or clinician and supervisor characteristics, such as EBT delivery experience, that might inform how supervision is conducted. Finally, this study advances existing knowledge by exploring the link between supervision approaches with clinicians’ delivery of TF-CBT, an EBT for posttraumatic stress, depression and disruptive behaviors among children exposed to traumatic events (20, 21). Trauma exposure is pervasive among youth, with nearly two-thirds of children in the United States experiencing a traumatic event before adulthood (22, 23). Treatment for trauma-exposed youth in routine practice settings often lack key evidence-based elements, notably the proportion of trauma-exposed youth receiving exposure has ranged from 14−22% (24). Clinicians have reported discomfort delivering exposure with trauma-exposed youth, which likely contributes to this gap in care (25). Supervision specifically focused on TF-CBT may improve the quality of care for trauma-exposed youth. While we focus on supervision for TF-CBT, the supervision techniques used are generalizable to other treatment modalities. Given this generalizability, this study holds promise for informing supervision implementation strategies that fit with how supervision is conducted in community-based workplaces and that are poised to influence clinician’s EBT delivery.

### 1.1. Current study

The current observational study uses an exploratory pattern-oriented approach (26) to examine whether distinct supervision approaches, reflecting varying patterns of supervision technique use, emerged among supervisors providing workplace-based supervision of TF-CBT in community mental health settings. We also examined whether emerging supervision approaches differentially predicted clinician’s delivery of EBT components. Data come from the first phase of a two-phase NIMH-funded study of workplace-based supervision (27). Phase 1 is a descriptive phase that characterizes supervision-as-usual among supervisors of clinicians delivering TF-CBT. Phase 2, which is not the focus of this study, tests the effect of two supervision packages on treatment fidelity and client outcomes.

We examined the presence of clusters of supervision techniques (i.e., supervision approaches). Next, we examined whether the dose of each supervision approach was related to TF-CBT delivery. TF-CBT components are summarized by the acronym “PRACTICE” and are grouped in three phases: (1) stabilization consisting of psychoeducation, parenting (for caregivers), relaxation, affective modulation, cognitive coping (i.e., PRAC), (2) trauma narration and processing consisting of the trauma narrative and cognitive processing (i.e., T), and (3) integration and consolidation including *in vivo* exposure, conjoint caregiver-child sessions, and enhancing safety (i.e., ICE). In this study, we focus on delivery of stabilization phase components and the trauma narrative. Specifically, we examine (1) whether the dose of each supervision approach a clinician received predicted the intensity with which they delivered components in the stabilization phase of TF-CBT, and (2) examine whether the dose of each supervision approach a clinician received predicted their delivery of the trauma narrative with youth.

## 2. Materials and methods

### 2.1. Participants

#### 2.1.1. Supervisors

Thirty-three supervisors were enrolled from 18 public mental health organizations in 23 offices in Washington state. This study includes data from 28 supervisors from 17 organizations who submitted recordings of individual supervision sessions. Three supervisors did not submit recordings after enrollment–two of whom left their organizations within 2 months–and two additional supervisors submitted group recordings that could not be coded. Seventy-two percent of organizations and 76.7% of the supervisors approached agreed to participate. *To* be eligible, supervisors were required to (a) have TF-CBT-specific training, provided through the Washington State EBT initiative and (b) be a current supervisor of two or more study-eligible clinicians. There were no exclusionary criteria. Previous comparisons between those who did and did not submit recordings demonstrated few significant differences. Supervisors who submitted recordings were slightly older (*M* = 44.4 vs. 37.8, *p* < 0.05), more likely to self-report that their primary theoretical orientation was cognitive-behavioral (75% vs. 0%, *p* < 0.05), and less likely to self-report that their primary orientation was family systems therapy (21 vs. 60%, *p* < 0.05) or art/play therapy (0 vs. 40%, *p* < 0.05) (12).

#### 2.1.2. Clinicians

Ninety-five clinicians were enrolled in the study, and data for 70 clinicians (74%) who were recorded in supervision sessions were included in the current analyses. Of the 125 clinicians approached, 76% agreed to participate. To be eligible, clinicians must have (a) been trained in TF-CBT through the EBT initiative, (b) provided TF-CBT to children or adolescents, (c) been supervised by a participating supervisor, (d) been employed at least 80% full-time equivalent, and (e) provided treatment in English (to allow for treatment coding). As previously reported, few significant differences emerged between clinicians who were and were not recorded. Clinicians who were recorded had provided psychotherapy for longer (*M* = 7.0 vs. 4.3, *p* < 0.05) and were less likely to have a degree in Marriage and Family Therapy (11% vs. 40%, *p* < 0.05) than those not recorded (12).

#### 2.1.3. Children and adolescents

Participants included 60 children and their guardians who received TF-CBT from an enrolled clinician being supervised by a participating supervisor. Of the 84 families approached, 71% agreed or were eligible to participate. Among non-participants, 50% were unreachable following referral from their clinician, 42% declined, and 8% were ineligible (e.g., age and clinical appropriateness). To be eligible, youth were required to be clients of a participating clinician. Inclusion criteria included: (a) age 6–17; (b) trauma history; (c) significant posttraumatic stress (PTS) symptoms; (d) live with a parent/legal guardian who is willing to participate in the study; (e) English-speaking; and (f) treatment approach is TF-CBT. Exclusionary criteria included (a) the youth having a pervasive developmental disorder or cognitive impairment and (b) parental serious mental illness.

#### 2.1.4. Participant sample per aim

Participant sample size for each aim is described in Table 1. Twenty-three supervisors supervised at least one clinician who enrolled a TF-CBT case and submitted at least one audio recorded TF-CBT session. Forty clinicians submitted at least one TF-CBT session. Among those, 34 submitted at least one file that incorporated a TF-CBT skills and stabilization-phase component (i.e., psychoeducation, relaxation, affective modulation, and/or cognitive coping). Among clinicians who did not submit a recording, some left their organization or did not have an eligible TF-CBT case. Among the 60 enrolled children, 49 had a recorded TF-CBT session, and 39 had a stabilization-phase session.

**TABLE 1:** Participant sample size in each aim.

Participant type	Aim 1: supervision styles	Aim 2: stabilization intensity^a^	Aim 3: trauma narrative delivery^b^
Supervisor	28	21	23
Clinician	70	34	40
Child	N/A	39	49

^a^Participants included those who had at least 1 audio file for the stabilization phase of treatment.

^b^Participants included those who had at least 1 audio file from any phase of treatment.

### 2.2. Procedures

Procedures were approved by the Washington State Institutional Review Board. The parent study builds on a Washington State EBT training initiative, currently in its 14th year, described elsewhere (12). The program includes training in TF-CBT, depression, anxiety, and behavior problems for public mental health organizations consisting of a 3-day in-person training and 6 months of consultation on applying treatment models with training cases for clinicians and supervisors. Monthly technical assistance calls and an annual 1-day supervisor training were made available to supervisors. Technical assistance and supervision training cover topics such as updates on the EBT initiative, sharing relevant research, and discussion and practice of supervision content and techniques. Only a subset of supervisors attended the voluntary technical assistance and training. Each organization had at least one supervisor complete the initiative expectations. Organizations were able to send trainees annually to address organizational growth and attrition.

Potential participants were identified via approaching organizations that had participated in the EBT initiative and still had at least one TF-CBT-trained supervisor in their organization. Senior leaders and supervisors were provided detailed study descriptions and informed consent was collected from interested supervisors. Participating supervisors identified potentially eligible clinicians among their supervisees. Clinicians were invited to participate in the study by the research team and informed consent was obtained. Enrolled clinicians were asked to introduce the study to caregivers of all youth who were potentially eligible to receive TF-CBT and who met the study inclusion and exclusion criteria. If families were interested, study staff proceeded with informed consent and recruitment. Supervisors audio recorded the portions of their supervision sessions that pertained to enrolled clinicians and their TF-CBT cases and submitted them weekly over the course of 1 year (October 2012–September 2013). Clinicians were asked to audio record all TF-CBT sessions with cases enrolled in the study for up to 6 months or until treatment termination, whichever came first. Clinicians labeled audio recordings with the primary treatment component for that session and recordings were date stamped. Organizations were compensated $3,000 for their participation at the end of the second phase of the RCT. Supervisors and clinicians were compensated $30 for completing the baseline survey. Guardians and youth were compensated $20 and $10 respectively, for completing the baseline survey.

#### 2.2.1. Session sampling

Two distinct sets of audio recordings were collected and coded in this study, TF-CBT supervision sessions and TF-CBT treatment sessions. In sum, 667 recordings of individual TF-CBT supervision sessions were received from 28 supervisors. Files shorter than 1 min were excluded (*n* = 29, 4.3%). Twenty-three recordings were coded per supervisor to balance the number of coded recordings among supervisors. When supervisors submitted greater than 23 recordings, stratified random sampling was used to select 23 recordings that were spread across time and clinicians. Eighteen supervisors submitted fewer than 23 recordings, and all were coded (*M* = 10.8; *SD* = 4.9; range 4–19). In total 438 (70%) were coded, three files were excluded from the current analyses due to missing codes, resulting in 435 coded supervision sessions. In total, 465 recordings of TF-CBT treatment sessions were received from 40 clinicians with 49 children. On average, clinicians submitted 8.61 recordings (*SD* = 4.59; Range = 1−19) during the 6-months any case was enrolled in the study. Two audio files were randomly selected per case, one audio file labeled with a skills/stabilization component, and one labeled as the trauma narrative. Among clients who were linked to a supervisor-clinician dyad who submitted supervision audio files, 39 had at least one stabilization session and 25 clients had at least one trauma narrative session.

#### 2.2.2. Intervention

Supervision was focused on the delivery of TF-CBT with trauma-exposed youth. TF-CBT is an EBT that applies principles of cognitive-behavioral therapy to address symptoms of posttraumatic stress, depression and disruptive behaviors among children exposed to traumatic events (20, 21). Given that in this study, treatment delivery was measured for 6 months, many clinicians did not make it to the integration and consolidation phase, and thus we focus on the stabilization and trauma narration phases. TF-CBT is a conjoint caregiver-child treatment, designed to be delivered in 12−16 sessions. Caregivers and children engage in parallel sessions for each treatment component and conjoint sessions, some of which involve the child and caregiver practicing skills together, the child sharing their trauma narrative, and other conjoint work as appropriate.

### 2.3. Measures

#### 2.3.1. Observer-rated supervision techniques

The intensity with which supervisors used 13 supervision techniques during supervision sessions was coded using the Supervision Process Observational Coding System (SPOCS). Development of the SPOCS is described elsewhere (12). The SPOCS includes 29 supervision strategies: 16 content areas and 13 techniques. The current study used data from only the coded supervision techniques, described in Table 2. Though developed for TF-CBT supervision coding, the SPOCS supervision techniques are likely applicable to supervision of other treatments. By excluding the TF-CBT supervision content, we maximize the generalizability of these findings to supervision of other treatment modalities.

**TABLE 2:** Supervision techniques, definitions and examples.

Supervision techniques	Definition	Example
Assigning additional training/learning	Supervisor clearly requests for clinician to obtain additional training or expertise for his/her own learning.	“There’s a chapter in the TF-CBT book on kids in foster care. Read that and see if you get any ideas for this client.”
Clinician behavioral rehearsal (in supervision)	Supervisor guides clinician through practicing effective use of a therapeutic skill/technique for a future session.	“Let me play your client. ‘But it’s too hard! I can’t talk about what happened’.” [Clinician role plays response]
Clinical suggestions	Supervisor gives specific suggestions and/or directions to clinician for a future session and/or about what clinician should have done in a past session.	“You said that last session the mom asked about what happens at court. I might have described the process or given her ideas for finding out more information.”
Didactic instruction	Supervisor provides information, teaches, and/or explains something to clinician via “lecture” or in a didactic style.	“Research shows PTSD symptoms can mimic hallucinations, such as thinking they can hear the offender talking.”
Elicitation	Supervisor uses questions to (a) encourage clinician thinking and planning for a subsequent session (vs. providing suggestions) or (b) help clinician evaluate their effectiveness in a past session.	“He’s really blaming himself for what happened. What do you think are some possible other ways to view the situation, given what you know about the case?”
Fidelity/adherence assessment	Supervisor and/or clinician discuss the topic of fidelity to TF-CBT or clinician’s progress through the model.	“Let’s review the TF-CBT components. Tell me which components you’ve completed and which you’re on now.”
Information gathering	Supervisor gathers information about the case, a past session, and/or clinician’s therapeutic/TF-CBT skill-level.	“When is the IEP meeting?”
Progress note review	Supervisor reviews the progress note with clinician or alludes to review that occurred before the supervision meeting.	“Let’s look at your case note for that session.”
Reviewing assigned suggestions/training	Supervisor specifically checks in about and/or asks about a suggestion, strategy, training, and/or recommendation from a past supervision session.	“It sounds like you didn’t sign up for the CBT + training like we talked about. What happened with that?”
Review of actual practice	Prior to or during supervision, the supervisor: (a) watched/listened to clinician’s session recording or (b) reviewed client work from a past TF-CBT session.	“Watching your tape, I noticed you did most of the talking. Why might that not be the most effective way to change the mother’s behavior with her child?”
Supervisor modeling	Supervisor models (i.e., enacts or demonstrates) a specific clinical skill or method of delivering a treatment component.	“You might say, ‘Hey, is there anything that happened in your past that guides how you deal with your daughter?”
Supportive listening	Supervisor reflects, validates, acknowledges, and/or praises clinician.	“Supervisor: “Sounds like a frustrating situation.”
Symptom monitoring	Supervisor and/or clinician discuss repeated use of standardized assessment measures to determine the symptom trajectory.	“So remind me, he’s at a 13 now, what was his score on the S when you started?”

#### 2.3.2. Coder training

Coders were six post-baccalaureate research assistants. They were first trained to reliably code TF-CBT component delivery (described below), given that study investigators assumed ability to code TF-CBT fidelity was a prerequisite for coding TF-CBT supervision. Coders attended a 2-day TF-CBT clinical training, completed a 10-h TF-CBT online course, read the TF-CBT treatment manual (20), and participated in didactic training in distinguishing components of the treatment model with a TF-CBT treatment developer, expert trainer (and study PI), and an experienced coder in prior TF-CBT studies. Coders were also trained to code supervision of TF-CBT. Coder training for fidelity and supervision coding consisted of similar activities, involving independent review of the respective manuals, didactic training, independent coding, group review of coded sessions, and joint listening to sessions when necessary to reach consensus. Three expert coders first coded 10 training files and came to consensus on their codes. Coders then independently coded 10 training files to ensure acceptable inter-rater reliability across group members and with the expert trainers. Coder training was complete once their individual ratings at the overall level met the threshold for inter-rater reliability, intra-class correlation coefficient (ICC) (2,1) ≥ 0.80 (28). If an individual content or technique item had an ICC (2,1) ≤ 0.60, coders were assigned additional review and practice. Coders were required to review the respective coding manuals monthly and to attend periodic booster trainings to maintain high reliability. Coders were also periodically provided with feedback on their inter-item reliability. Audio recording files were randomly assigned to each coder.

#### 2.3.3. Coding procedures

Trained coders rated technique occurrence in 5-min intervals (low, medium, or high), yielding intensity scores for techniques for each session (0–6 range; 0: *non-occurrence*; 1–2: *low*; 3–4: *medium*; 5–6: *high intensity*). For instance, a low-intensity rating of supportive listening would be given for a limited number of supervisor non-specific acknowledgments or general praise (e.g., “nice work;” “that sounds hard”), while a higher score would be given if the supervisor provided more frequent and explicit support, validation, or praise (e.g., “…sounds like a tough session; still, you did a really nice job getting this super anxious kid to feel comfortable talking about his sexual abuse. I am impressed.”). Use of audio recordings restricted coding to verbal behavior.

#### 2.3.4. Coding reliability

Twenty-four percent of the coded supervision session recordings were coded by multiple coders to assess interrater reliability. The overall group average ICC was ICC(2,6) = 0.87, suggesting excellent reliability (28). Individual coders had excellent ICCs of 0.84 or higher. At the item level, ICCs ranged from 0.19 to 0.95. Of note, only 2 of the individual 13 technique item-level codes were below 0.60: *Assigning Additional Training/Learning* and *Reviewing Assigned Suggestions or Trainings*. Given their poor reliability and low incidence (see Table 3), these two techniques were excluded from the analyses (see Ref. 12, for more details).

**TABLE 3:** Supervision technique descriptive statistics and correlation matrix.

Technique	*M* (*SD*)	Min–Max	Occurred (%)	1	2	3	4	5	6	7	8	9	10	11	12	13
Assign training/learning^a^^,^^b^^,^^c^	0.06 (0.33)	0-3	4%	—												
Clinical suggestions	2.71 (1.57)	0-6	88%	0.02	—											
Behavioral rehearsal^c^	0.26 (0.59)	0-6	17%	0.04	0.13	—										
Didactic instruction	2.81 (1.34)	0-6	94%	0.15	0.56	0.12	—									
Elicitation	0.86 (1.06)	0-5	51%	-0.03	0.28	0.28	0.20	—								
Fidelity monitoring	1.72 (1.59)	0-6	67%	-0.01	0.26	0.05	0.11	0.07	—							
Information gathering	2.87 (1.21)	0-5	97%	0.01	0.24	-0.03	0.20	0.20	0.20	—						
Modeling	0.93 (1.23)	0-5	46%	0.04	0.54	0.29	0.47	0.28	0.13	0.09	—					
Progress note review^b^^,^^c^	0.17 (0.75)	0-5	6%	-0.04	0.03	0.02	-0.04	0.04	0.12	0.12	-0.04	—				
Review practice^b^^,^^c^	0.13 (0.59)	0-5	5%	-0.04	0.05	0.01	-0.03	0.01	-0.01	0.04	0.04	-0.05	—			
Review suggestions/learning^a^^,^^b^^,^^c^	0.06 (0.30)	0-3	5%	0.08	0.04	-0.07	0.01	-0.01	0.05	0.02	0.02	-0.02	-0.03	—		
Supportive listening	3.93 (1.01)	0-6	100%	0.04	0.17	0.06	0.26	0.23	0.14	0.43	0.16	-0.01	0.07	0.06	—	
Symptom monitoring^c^	0.60 (1.17)	0-6	26%	-0.07	0.15	-0.05	-0.07	0.11	0.22	0.10	0.01	0.31	0.01	0.03	0.05	—

^a^Excluded due to poor reliability.

^b^Excluded due to low occurrence.

^c^Excluded due to low covariance with other techniques.

#### 2.3.5. Observer-rated treatment delivery

The presence and intensity with which TF-CBT components were delivered were coded using a TF-CBT specific version of the Therapeutic Process Observational Coding System for Child Psychotherapy (TPOCS-S; 29). The TF-CBT TPOCS-S (30) was developed using Garland’s TPOCS-S (31) as a basis, incorporating 10 TF-CBT content area items (e.g., relaxation, trauma narrative) and 2 general items (i.e., assessment, other topics/crisis or case management). These 12 content items and 13 therapeutic techniques (e.g., assign/review homework, Socratic questioning) were coded for intensity ranging from 0 to 6 (0: *non-occurrence*; 1–2: *low*; 3–4: *medium*; 5–6: *high extensiveness*). Extensiveness, or intensity, reflects two related dimensions, thoroughness, and frequency. Coded data were used to characterize two aspects of treatment delivery: (1) intensity of *stabilization* phase delivery and (2) *trauma narrative* delivery. Stabilization phase intensity was calculated using the highest intensity score for any stabilization phase content item in the session, yielding an intensity score ranging from 1 to 6. Two audio files were randomly selected per case, capturing the stabilization phase of TF-CBT treatment involving the psychoeducation, relaxation, affective modulation, and/or cognitive coping components. The current study uses only individual child sessions to assess stabilization phase intensity.

The occurrence of the trauma narrative was determined using the audio recording labels submitted by clinicians and then listening to the content to confirm the trauma narrative was delivered. Clinicians labeled audio files with the primary session component. For quality assurance, coders listened to the audio file to ensure inclusion of the trauma narrative component. If there was no discussion of the trauma narrative in the audio file, the file was relabeled to document the primary TF-CBT component. If a clinician never submitted an audio file labeled “trauma narrative” or no other session was deemed to include the trauma narrative based on coder review, they were considered to have not delivered this component.

#### 2.3.6. Coding reliability

Forty eight percent of coded TF-CBT sessions recordings were coded by two or more coders and interrater reliability was computed to ensure every coder maintained reliability standards. Four waves of interrater reliability testing were conducted to protect against coder drift. Using absolute agreement, single measures, and two-way random effects models, ICCs (2,2) were equal to or greater than 0.70 for each of these waves, and averaged 0.76, suggesting excellent reliability (28). At the item-level, ICCs for the stabilization and trauma narration content ranged from 0.77 to 0.84.

#### 2.3.7. Participant characteristics

Clinicians, supervisors and guardians of youth completed baseline surveys about their demographic characteristics.

#### 2.3.8. Youth PTS severity

The UCLA Posttraumatic Stress Disorder Reaction Index is a 20-item measure that was used to assess youth PTS severity (32). Following measure guidelines guidance, we used a severity cutoff score of 21 and higher or algorithm scoring for likelihood to meet diagnostic criteria. This measure demonstrates good convergent validity and good-to-excellent test-retest reliability, with a Cronbach’s α in the range of 0.90 of the entire scale (33).

#### 2.3.9. Dose of supervision approach

Cluster-based analysis (described below) was used to identify clusters characterizing how intensely supervisors used the set of supervision techniques. Each cluster was conceptualized as a supervision approach. Because supervision approach was specific to a supervision session, the dose of supervision approach was calculated as the proportion of supervision sessions during which a clinician received each supervision approach.

### 2.4. Analytic plan

#### 2.4.1. Aim 1: explore the presence of clusters of supervision techniques (i.e., supervision approaches)

Agglomerative hierarchical cluster analysis utilizing minimum variance method (i.e., Wards method) with a Canberra distance measure (34) was conducted to identify clusters of supervision technique use across eleven supervision techniques (i.e., supervision approaches). Cluster analysis is a data reduction technique that aims to increase within-group homogeneity and between-group heterogeneity to identify homogenous subgroups based on shared properties (35, 36). Data were mean centered (37) prior to analysis. The balance of the overall clustering structure was assessed using the agglomerative coefficient and appropriateness of the hierarchical structure imposed by clustering was evaluated using the cophenetic correlation (37). Overall clustering structure and cluster-specific fit indices were inspected to aid in cluster selection.

##### 2.4.1.1. Variable selection approach

Inclusion of unnecessary or non-informative variables can add noise and obscure true clusters (38, 39). Noise variables could include supervision techniques that (1) do not discriminate between supervision approaches due to low variability in their use or (2) that do not covary with other techniques. For instance, passive-supportive techniques (e.g., supportive listening) may poorly discriminate between supervision approaches because they are commonly used with high intensity compared to other techniques variable usage that may capture meaningful differences between supervision approaches. Therefore, variable descriptive statistics and correlations were examined before conducting cluster analysis to omit potentially non-informative techniques. Techniques were omitted if they rarely occurred (i.e., ≤5% of supervision sessions) or did not show evidence of moderate covariance with other techniques, defined as a correlation of *r* ≥ 0.20 with at least 30% of techniques. Upon conducting hierarchical cluster analysis, if fit indices did not meet recommended thresholds described below, a data reduction approach was used to eliminate supervision techniques that precluded the identification of stable and valid clusters. This approach was informed by theory and empirical evidence for the most important supervision techniques in shaping clinical practice and by observed separation in supervision techniques.

##### 2.4.1.2. Cluster selection

Solutions were evaluated by inspecting the dendrogram and agglomerative coefficient scree plot to aid in selection of the demarcation point indicating an appropriate cluster solution. We examined fit indices reflecting (a) cluster size, (b) cluster separation, and (c) homogeneity of clusters when determining the most appropriate solution (37). These included between-cluster separation and within-cluster homogeneity using the Silhouette Width (SW) and Dunn Index (DI) (37). Cluster stability was assessed to evaluate the meaningfulness and potential for spurious clusters. A non-parametric bootstrapping approach (40) was used by which cluster analysis was repeated on random samples with replacement from our data (*N* = 435, B samples = 1000). The mean maximum Jaccard coefficient, a measure of the similarity of two sets, was calculated to estimate cluster stability.

##### 2.4.1.3. Cluster interpretation

Resulting cluster assignments represent the supervision approach used by a supervisor, with a given clinician, during a single supervision session. Clustering at this level allowed us to characterize variability in a supervisors’ use of a supervision approach with a particular clinician and across all of their clinicians. This was calculated as: (1) the proportion of sessions a supervisor was assigned to a particular cluster with a *specific* clinician (i.e., supervisor-clinician level summary) and (2) the proportion of sessions for which a supervisor was assigned to a particular cluster across *all* of their clinicians (i.e., supervisor-level summary). The supervisor-clinician level summary allowed for the characterization of the “dose” of each supervision approach a clinician received across their supervision sessions, which was used as the predictor of TF-CBT delivery.

#### 2.4.2. Aim 2: dose of supervision approach predicting TF-CBT stabilization component intensity

We examined the relation between “dose” of each supervision approach and clinicians’ delivery of stabilization-phase components using a generalized estimating equation (GEE) model with an exchangeable correlation structure, accounting for the nesting within client. While a four-level multilevel model accounting with random-intercepts for the supervisor, clinician, and client would be most appropriate for this analysis, each higher level of clustering had a high frequency of clusters with *n* = 1 (e.g., most clinicians with a single client), precluding the use of multilevel modeling due to model non-convergence (41). We controlled for youth PTS severity as symptom severity may influence clinicians’ EBT delivery. To assess for model sensitivity, we randomly sampled our data to eliminate nesting and replicated the analysis using a linear regression model to compare the results with and without accounting for nesting.

#### 2.4.3. Aim 3: dose of supervision approach predicting trauma narrative delivery

A logistic regression model was estimated to examine the relation between “dose” of each supervision approach and trauma narrative delivery. As with aim 2, a three-level mixed effects logistic regression model with random-intercepts for clinician and supervisor would be most appropriate to account for the nesting but low cluster sample sizes precluded the use of this model. We again controlled for youth PTS severity, as we predicted that higher PTSD severity may reduce the likelihood that a clinician is able to deliver the trauma-narrative within the first 6 months of treatment. To assess for model sensitivity, we randomly sampled our data to eliminate nesting and replicated the analysis to compare the results with and without accounting for nesting.

## 3. Results

Table 4 presents demographic information for participants. Supervisors and clinicians were predominantly female (supervisor 64.3%, clinician 87.1%), White (supervisor 92.9%, clinician 88.6%), held a Master’s degree (supervisor 92.9%, clinician 88.6%), and endorsed a primary theoretical orientation of cognitive-behavioral (supervisor 75.0%, clinician 64.3%). Youth were predominantly female (61.7%), White (50.0%), from a household with an income less than $25,000 (53.3%) and receiving Medicaid-funded services (88.3%). The average age of youth was 11.5 (*SD* = 2.1) and average PTS severity score was 30.1 (*SD* = 13.9).

**TABLE 4:** Participant demographic and baseline characteristics.

	Supervisors (*n* = 28)		Clinicians (*n* = 70)		Clients (*n* = 60)	
	***n***	**%**	***n***	**%**	***n***	***%***
Female	18	64.3%	61	87.1%	37	61.7%
Male	10	35.7%	9	12.9%	23	38.3%
Race/ethnicity						
American Indian/Alaska Native	1	3.6%	1	1.4%	1	1.7%
Asian	1	3.6%	3	4.3%	0	0.0%
Black/African American	0	0.0%	0	0.0%	1	1.7%
Hispanic or Latinx	0	0.0%	8	11.4%	18	30.0%
White (non-Hispanic)	26	92.9%	62	88.6%	30	50.0%
Multiracial	0	0.0%	1	1.4%	10	16.7%
Other (not specified)	0	0.0%	1	1.4%	0	0.0%
Household income						
<$25,000	–	–	–	–	32	53.3%
25,000–49,999	–	–	–	–	20	33.3%
50,000–74,999	–	–	–	–	2	3.3%
75,000+	–	–	–	–	6	10%
Medicaid recipient	–	–	–	–	53	88.3%
Education level						
Bachelor’s	0	0.0%	5	7.1%	–	–
Master’s	26	92.9%	62	88.6%	–	–
Doctoral	2	7.1%	3	4.3%	–	–
Academic degree/background						
Marriage and family therapy	5	17.9%	8	11.4%	–	–
Psychology	3	10.7%	4	5.7%	–	–
Social work	11	39.3%	19	27.1%	–	–
Counseling psychology	9	32.1%	28	40.0%	–	–
Other	0	0.0%	11	15.7%	–	–
Primary theoretical orientation						
Cognitive-behavioral	21	75.0%	45	64.3%	–	–
Family systems	6	21.4%	7	10.0%	–	–
Solution-focused	1	3.5%	3	4.3%	–	–
Humanistic	0	0.0%	4	5.7%	–	–
Psychodynamic	0	0.0%	7	10.0%	–	–
Play therapy	0	0.0%	3	4.3%	–	–
Art therapy	0	0.0%	1	1.4%	–	–
Licensed	27	96.4%	36	51.4%	–	–
	***M***	***SD***	***M***	***SD***	***M***	***SD***
Age	44.4	10.4	38.0	11.5	11.5	2.1
PTS severity	–	–	–	–	30.1	13.9

### 3.1. Aim 1: examining the presence of clusters of supervision techniques

Descriptive statistics and the correlation matrix for the supervision techniques are included in Table 3. Supportive listening (100%), information gathering (97%), didactic instruction (88%) and clinical suggestions (88%) were used most often across supervision sessions. Techniques used least often included assigning training or learning (4%), review of suggestions or learning (5%), review of practice (5%), and progress note review (6%). Behavioral rehearsal, progress note review, review of actual practice, and symptom monitoring were omitted due to low occurrence or low covariance with other techniques to avoid inclusion of non-informative techniques. As described previously, assigning training/learning and reviewing suggestions and learning were omitted due to poor coding reliability. The remaining seven techniques were included in the hierarchical cluster analysis.

**FIGURE 1:**
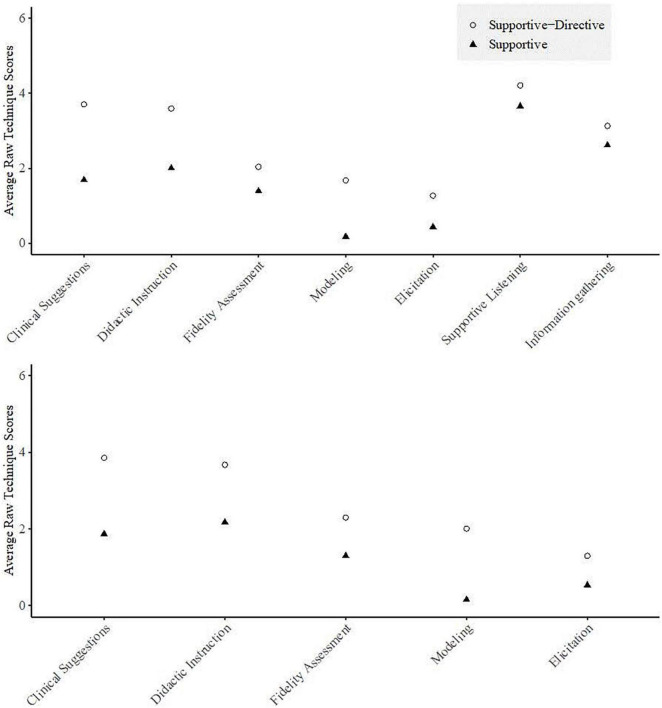
Average technique scores for the 7 supervision techniques **(top)** and 5 supervision techniques **(bottom)**.

Results for the overall cluster analysis showed a high agglomerative coefficient (0.98) but low cophenetic correlation (0.49), suggesting a strong clustering structure but poor fit between the original data and the clustering. Inspection of the dendrogram and agglomerative coefficient scree plot suggested a two-cluster solution best fit the data. Cluster 1 demonstrated good stability per the Jaccard coefficient, however cluster 2 was unstable (see Table 5). Given the low cophenetic correlation and instability of cluster 2, the average separation of each supervision technique was inspected to identify techniques that may be reducing fit indices and stability to inform our data reduction approach. Inspection of the average technique scores (see Figure 1) for observations grouped into cluster 1 compared to those in cluster 2 revealed limited separation of fidelity assessment (*M1* = 1.39, *SE* = 0.10; *M2* = 2.04, *SE* = 0.12) supportive listening (*M1* = 3.65, *SE* = 0.07; *M2* = 4.21, *SE* = 0.07), and information gathering (*M1* = 2.61, *SE* = 0.09; *M2* = 3.13, S*E* = 0.08). We aimed to retain techniques with the most theoretical and empirical support that the extent to which they are used in supervision would impact EBT delivery. Empirical evidence suggests that supervision that includes a focus on adherence to treatment principles predicts greater clinician treatment adherence (2). Therefore, fidelity assessment was retained despite its low separation. In contrast, while supportive listening is a foundational tool for developing supervisory alliance, the empirical validity of supervisory alliance has been described as “tentative” at best (42). Information gathering, while likely a necessary technique in all supervision sessions, is too non-specific to differentiate between supervision or its impact on clinical practice.

**TABLE 5:** Hierarchical cluster analysis fit and stability indices.

Techniques	Cluster n^a^	Agglomerative coefficient	Cophenetic correlation	Silhouette width^b^	Dunn index^c^	Jaccard coefficient^d^
7	2	0.98	0.49	0.21	0.11	0.77; 0.71
5	2	0.99	0.62	0.29	0.17	0.76; 0.79

^a^Cluster n refers to the number of clusters in each cluster-solution.

^b^Silhouette Width calculates the average distance between clusters. Values closer to 1 indicate well clustered observations, negative values indicate inaccurate clustering.

^c^Dunn Index assesses cluster compactness and separation. Larger values indicate more compact, well-separated clusters.

^d^Jaccard Coefficient estimates cluster stability. Values above 0.75 are considered stable.

The results for the 5-technique cluster analysis showed a high agglomerative coefficient (0.98) and an improved cophenetic correlation (0.62), suggesting an acceptable cluster structure and fit with the original data. A two-cluster solution was selected based on inspection of the dendrogram and agglomerative coefficient scree plot. Fit indices were improved relative to the 7-technique cluster analysis and clusters demonstrated good stability (see Table 5). Figure 1 shows that relative to cluster 2, cluster 1 had higher intensity scores across all techniques, with the greatest differences in the techniques clinical suggestions, modeling of a particular skill or method and didactic instruction. Cluster 1 demonstrated moderate intensity use of clinical suggestions (*M* = 3.86, *SD* = 0.08) and didactic instruction (*M* = 3.67, *SE* = 0.06) while cluster 2 demonstrated low intensity use of all techniques. Although supportive listening did not reliably distinguish between clusters, this technique was used with the highest intensity across both clusters. Therefore, we term cluster 1 *supportive*–*directive* supervision to reflect the higher intensity use of techniques intended to direct clinicians practice (i.e., clinical suggestions and didactic instruction) and term cluster 2 *supportive* supervision. *Supportive*–*directive* supervision was characterized by longer supervision sessions (in minutes) (*M* = 27.07, *SD* = 14.81, range = 5–72) than *supportive* sessions (*M* = 19.30, *SD* = 13.74, range 5–60), *t*(358.26) = −5.44, *p* < 0.001. This corresponds to spending an average of 15.39 (*SD* = 8.33) minutes per client during *supportive*–*directive* supervision and 10.71 (*SD* = 8.62) minutes per client in *supportive* supervision.

Across all supervision sessions (session-level), 42.17% were assigned to the *supportive*–*directive* cluster and 57.83% were assigned to the *supportive* cluster, suggesting that supervision sessions were more commonly characterized by less intensive use of the five supervision techniques. At the supervisor level, 60.7% of supervisors demonstrated a tendency to use a mix of both supervision approaches across sessions, defined as use of *supportive*–*directive* supervision in 26%–74% of sessions. In contrast, 14.30% of supervisors showed a tendency toward consistently using *supportive*–*directive* supervision (i.e., ≥75% of sessions) and 25% showed a tendency toward consistently using *supportive* supervision (i.e., ≥75% of sessions). Examining the consistency of supervision approach use at the clinician level demonstrated that supervisors tended to use a more consistent supervision approach when working with a particular clinician. Specifically, 20% of supervisors showed a tendency toward often using *supportive*–*directive* supervision (i.e., ≥75% of sessions), 41.40% showed a tendency toward often using *supportive* supervision (i.e., ≥75% of sessions), and 38.60% pivoted between the two approaches (i.e., using *supportive*–*directive* supervision in 26%–74% of sessions). Findings suggests that while supervisors most often used both supervision approaches, they showed more consistency in their supervision approach for a given clinician.

### 3.2. Aim 2: dose of supervision approach predicting TF-CBT stabilization component intensity

All clients (100%) included in aim 2 received components from the stabilization phase, including psychoeducation, relaxation, affective modulation, or cognitive coping, and the average intensity was 4.06 (*SD* = 1.13), reflecting ‘medium intensity’ of these treatment elements. This suggests that, on average, clinicians delivered stabilization phase elements with relative thoroughness and frequency, as intensity scores of 5–6 are not expected to occur commonly. The results of the GEE and multiple regression models (Table 6) were largely comparable, suggesting minimal bias due to ignoring nesting. Given that the relationship between *supportive*–*directive* supervision and stabilization component scores was only marginally statistically significant in the GEE model, we retain the interpretation from the multiple regression sensitivity analysis. The regression analysis suggests that neither the clinicians’ dose of *supportive*–*directive* supervision nor a client’s baseline PTSD severity were statistically significantly related to the intensity with which clinicians delivered components in the stabilization phase of TF-CBT, *B* = 0.71, *t*(36) = 1.21, *p* = 0.24.

**TABLE 6:** Model of *supportive–directive* supervision predicting trauma-focused cognitive behavioral therapy stabilization scores.

Generalized estimating equation^a^	*B*	Robust *SE*	95% *CI*	Robus*t Z*	*P*
Intercept	4.05	0.34	[3.38, 4.72]	11.87	<0.01
Dose of *supportive–directive* supervision	0.62	0.32	[−0.01, 1.25]	1.92	0.05
PTSD severity	−0.01	0.01	[−0.02, 0.01]	−0.86	0.39
**Multiple regression^b^**	***B***	***SE***	**95% *CI***	***t***	***p***
Intercept	4.02	0.56	[2.89, 5.15]	7.21	<0.01
Dose of *supportive–directive* supervision	0.71	0.58	[−0.48, 1.89]	1.21	0.24
PTSD severity	−0.01	0.02	[−0.03, 0.03]	−0.11	0.91

^a^Full sample including sessions (n = 62) nested within clients (n = 39) and clinicians (n = 34).

^b^Sensitivity analysis with a random sample of 1 session per client (n = 39) to eliminate nesting.

### 3.3. Aim 3: dose of supervision approach predicting trauma narrative delivery

Thirty-one clients (63%) received the trauma narrative component of TF-CBT within the first 6 months of treatment. Among these clients, the average intensity score for the trauma narrative component was 4.07 (*SD* = 1.04), reflecting ‘medium intensity’ of this treatment element. The results (see Table 7) suggest that when clinicians received *supportive*–*directive* supervision in all supervision sessions, the odds of a clinician delivering the trauma narrative was 18.46 times higher than a clinician who received *supportive* supervision in all supervision sessions (OR = 19.92; CI = 1.92–196.82). Notably, the confidence interval was quite large, suggesting a statistically significant yet imprecise estimate. This trend was also supported when re-estimating the model using a random sample of clients to eliminate nesting. Youth PTSD severity showed a negative relationship with the odds of receiving the trauma narrative, however, this trend was only marginally statistically significant in the initial model and was not supported in the sensitivity analysis.

**TABLE 7:** Model of *supportive–directive* supervision predicting trauma narrative delivery.

Logistic regression^a^	*B*	Exp(*B*)	95% CI	*SE*	*z*	*p*
Intercept	1.26	3.53	[0.51, 24.48]	0.99	1.28	0.20
Dose of *supportive–directive* supervision	2.97	19.46	[1.92, 196.82]	1.18	2.51	0.01
PTSD severity	−0.05	0.95	[0.90, 1.01]	0.03	−1.79	0.07
**Logistic regression^b^**	***B***	**Exp(*B*)**	**95% CI**	***SE***	***z***	***p***
Intercept	0.84	2.32	[0.28, 19.19]	1.08	0.78	0.44
Dose of *supportive–directive* supervision	3.22	25.03	[199, 315.11]	1.29	2.49	0.01
PTSD severity	−0.04	0.96	[0.91, 1.02]	0.03	−1.38	0.17

^a^Full sample including clients (n = 49) nested within clinicians (n = 40).

^b^Sensitivity analysis with a random sample of 1 client per clinician (n = 40) to eliminate nesting.

## 4. Discussion

This study aimed to advance the limited knowledge of supervision methods used in workplace-based supervision to support clinicians delivering an EBT. We took an exploratory approach to examine patterns of supervision technique use that could inform the development of future supervision-focused implementation strategies. Critiques of pattern-oriented approaches, such as cluster analysis, include the risk of producing clusters that do not exist or that lack external validity (43). To address this, we took multiple steps to validate the results including resampling methods to examine the internal reliability of results and examining the predictive validity of the clusters in predicting observed clinician practice. Nonetheless, findings are exploratory and are intended to inform hypothesis generation for future research. In the following sections, we discuss our findings and considerations in their interpretation, situate our findings within the broader literature, and propose future directions.

Didactic instruction was among the highest intensity techniques used in both clusters, although only *supportive-directive* sessions used didactic instruction with moderate intensity on average. While didactic instruction is a common technique used in training (6), supervision (15), and consultation (44), studies evaluating training efforts demonstrate that didactic instruction without experiential learning techniques is not sufficient to impact clinician’s behavior and client improvement (6). S*upportive*–*directive* supervision incorporated experiential learning through the use of modeling, but this was notably absent in *supportive* supervision. Both approaches include low intensity fidelity assessment, although *supportive*–*directive* supervision included over a one-point higher average intensity. It is not entirely clear how meaningful a one-point difference in fidelity assessment may be in impacting clinician’s EBT delivery, as most studies that examine this association do not provide descriptions of technique dosage. One study found a 12% increase in therapist EBT adherence when comparing supervisors with the highest and lowest focus on adherence in supervision (2).

Empirical research on clinical suggestions and elicitation is lacking, however, theoretical perspectives provide some insight into how supervisors can use these techniques to support clinician learning. *Supportive*–*directive* supervision included moderately intensive clinical suggestions and low intensive elicitation, whereas *supportive* supervision included low intensive clinical suggestions and essentially no elicitation. Theory suggests that effective cognitive-behavioral supervision can support clinician learning through the use of scaffolding strategies (45), specifically through the use of asking questions. In the current study, elicitation involved a supervisor asking questions to (a) encourage and elicit clinician thinking and planning for a subsequent session and/or (b) to help the clinician to evaluate their own effectiveness in a previous session. James et al. (45) suggest questioning can be used to help the supervisor understand the clinician’s knowledge and to facilitate the clinician’s learning by eliciting their own ideas and providing feedback or clinical suggestions.

We anticipated approaches that demonstrated a clear preferential use of techniques, such as distinctions in techniques that serve similar functions but take on different forms. For instance, supervisors intending to engage clinicians in experiential learning could demonstrate preferential use of behavioral rehearsal or modeling. Instead, results more closely reflect a high and low intensity approach, although the magnitude of the difference in technique intensity did differ. Those techniques that may serve overlapping functions, including behavioral rehearsal, symptom monitoring, review of practice, and progress note review, were rarely used and tended to show low covariance with other techniques. Moreover, *supportive*–*directive* supervision, the high intensity approach, was longer in duration which raises the possibility that supervision length may account for these differences in approach and subsequently clinicians’ TF-CBT delivery. Alternatively, there may be an interaction between clinicians expressed needs and client issues that elicit supervisors to engage in *supportive*–*directive* supervision and devote additional time to supervision. Distinguishing between the influence of supervision time and technique use will be an important future step to inform the targets of supervision-focused implementation strategies.

The techniques that were nearly absent can inform an agenda of formative work to improve their uptake and usability in a supervision-focused implementation strategy to target EBT delivery. Among these techniques, three are considered to be ‘gold standard’ supervision techniques: behavioral rehearsal, review of practice, and symptom monitoring, which were not included in the cluster analysis due to a lack of evidence that they could show meaningfully distinct patterns of use. Their limited use may reflect attitude and feasibility challenges that would need to be addressed to increase their use. Nationally, 62% of providers reported never using standardized progress measures for symptom monitoring (46), citing both feasibility and ideological challenges (e.g., time, resource limitations, and case appropriateness) (47–49). Feasibility and efficiency challenges have also been raised for review of practice, due to the time and resource constraints and lack of fit with practice norms (50). While behavioral rehearsal is a more feasible technique (51), limited use has also been demonstrated during supervision and consultation (18, 44) and may reflect clinician discomfort engaging in behavioral rehearsals (44). Future implementation strategies intending to incorporate use of these gold standard techniques in supervision will likely require efforts to improve their feasibility and comfort with their use. Possible solutions include user-centered design methods to improve the design of the techniques to fit the local context (52), habituation techniques to decrease clinician discomfort with techniques (53), and targeting norms and attitudes to increase uptake (54).

We also detected patterns in the consistency with which supervisors engaged in a particular approach. When working with a specific clinician, supervisors demonstrated a greater degree of consistency in using either *supportive*–*directive* or *supportive* supervision, while they tended to engage in both supervision approaches across their clinicians. This could be a function of supervisors tailoring their supervision approach to fit the clinician, the clinician eliciting a certain approach through questions and issues raised, or a mix of the two. While supervisors often ascribe to a particular theoretical model of supervision (55), there has long been a recognition that supervision needs to be tailored to the developmental stage of the supervisee and that supervisees play an active role in shaping supervision (56). An important future direction is to explore characteristics of the setting, supervisor, clinician, and session that may influence the selection or elicitation of a particular approach. Related studies have largely failed to identify clinician or supervisor characteristics that influence practice, but have found that either the organizations’ climate toward EBTs (57, 58) or being situated within a private vs. public agency (18) influence supervision practice. Nonetheless, numerous factors could plausibly influence supervisors’ technique use. For instance, supervisors with training in and exposure to ‘gold standard’ techniques may be more likely to use them or clinicians demonstrating less experience with or knowledge of an intervention might elicit more directive and experiential techniques. Understanding the drivers of supervision technique use can inform what approaches would be most influential in shaping supervision. Our future work will explore multilevel factors–ranging from characteristics of the supervision session those of the supervisor–that influence supervision approaches.

While receiving a higher proportion of *supportive*–*directive* supervision did not impact the quality with which clinicians delivered stabilization phase components, it did impact whether they delivered imaginal exposure. Past studies of workplace-based supervision have established a link between supervision and clinician treatment adherence (1) and treatment competency (17). However, some of the supervision techniques in those studies were largely absent in the current study. For instance, Martino et al. (17) had supervisors provide corrective feedback based on review of actual practice and skills coaching using behavioral rehearsal. In our study, review of actual practice and behavioral rehearsal were nearly non-existent. Schoenwald et al. (2), found that supervision that attended to clinicians’ adherence to Multisystemic Therapy (MST) through discussions of MST assessment and intervention strategies predicted greater clinician adherence to MST. Although *supportive*–*directive* supervision included higher intensity use of fidelity assessment, the dose of fidelity assessment may not have been high enough to impact stabilization phase intensity. Alternatively, stabilization skills may be delivered with high intensity regardless of supervision practices. Most clinicians in this sample endorsed a CBT theoretical orientation, and developing coping skills are common components in CBT for traumatic stress and anxiety (59). Clinicians delivering CBTs often express a preference for treatment components focused on coping skills over exposure-based components (60) and exposure has been rated as the most difficult strategy to implement among CBT strategies (60). This preference and familiarity with treatment components that develop coping skills may diminish the importance of supervision in developing competency in these components. In contrast, the higher intensity technique use in *supportive*–*directive* supervision may have acted on clinicians’ established discomfort with exposure and perceptions of exposure as harmful (25) in several ways. For instance, supervisors use of modeling in *supportive*–*directive* supervision may have improved clinician’s comfort with delivering imaginal exposure. Fidelity assessment, including discussion of treatment pacing, keeping the treatment brief, and the client’s ability to move on to the next component, may have facilitated more timely pacing through the treatment model and alerted supervisors when a clinician was delayed in progressing to the trauma narrative. Future research could extend our work by examining the mechanisms through which this supervision approach impacts exposure delivery.

The longer total supervision session time and time per case in the *supportive*–*directive* relative to *supportive* approach warrant further exploration. Instances of 5-min supervision sessions being characterized as *supportive*–*directive* suggests that brief supervision sessions do not preclude *supportive*–*directive* supervision, yet there was a clear trend of *supportive*–*directive* supervision sessions being longer. The literature would benefit from future research that explores the interplay between time and supervision approaches. Clinicians in routine mental health settings often carry large client caseloads, and estimates suggest the time to discuss each case ranges from 5 to 12 mins (12, 61). Most clinicians and supervisors endorse wanting more time in supervision to spend on functions that are most relevant to EBT (10). Exploring creative ways to maximize either the total time spent in supervision or how time is spent when supervision is brief would be an important contribution to workplace-based supervision.

This study had a number of strengths. First, this study used coded supervision sessions and coded TF-CBT sessions, providing high-quality measures of supervision techniques and TF-CBT fidelity. The use of coded measures also eliminated common method variance that is present in studies that rely solely on self-report measures. Among the existing literature on supervision, much of it has examined supervision in the context of studies utilizing external Doctoral-level supervisors or consultants. This study focused on workplace-based supervision and delivery of treatment in community mental health settings, by clinicians employed in these settings, increasing the external validity of the study findings. The use of a cluster approach allowed for the characterization of supervision across a combination of various supervision techniques.

Several limitations should also be noted. First, supervision sessions were of TF-CBT cases and may not reflect supervision of other treatments. However, the average use of techniques in our study were similar to a study describing supervision of various treatments (18), supporting generalizability. Second, the sample was drawn from organizations that participated in a EBT initiative and supervisors endorsed a predominantly CBT orientation, potentially skewing the supervision approaches that emerged in this study. Moreover, the results of our original cluster analysis did not produce stable results. Our data-reduction approach poses the risk of overfitting the clusters to our data, underscoring the exploratory nature of these results. However, convergent validity of the resulting clusters was also supported through the significant associations with clinician’s trauma narrative delivery. Finally, we were unable apply a classification model that accounts for nesting, such as multilevel latent class analysis, given the small sample sizes at the group level (62), possibly biasing the clustering results.

## 5. Conclusion

To our knowledge, this is the first study to examine supervision approaches based on coded supervision techniques used to support clinician EBT delivery in community mental health. This study identified two approaches, *supportive*–*directive* and *supportive* supervision, and their use had an influence on whether or not clients received a trauma narrative, an important component of TF-CBT. Findings contribute to the characterization of workplace-based supervision and provide a starting point for examining approaches that shape EBT delivery. Our future directions include exploring drivers of supervision technique use and we encourage additional investigations in this area to inform evidence-based approaches to shape supervision practices.

## Data availability statement

The raw data supporting the conclusions of this article will be made available by the authors, without undue reservation.

## Ethics statement

The studies involving human participants were reviewed and approved by the University of Washington Institutional Review Board. Written informed consent to participate in this study was provided by the participants’ legal guardian/next of kin.

## Author contributions

RM, SD, and MP contributed to the conception and design of the study. RM performed the statistical analysis and wrote the first draft of the manuscript. MP contributed to the design of the analysis. RA wrote the sections of the manuscript. All authors contributed to manuscript revision, read, and approved the submitted version.
